# A Treatment Decision Model for Cutaneous Squamous Cell Carcinoma Based on Bayesian Networks

**DOI:** 10.3390/cancers18040704

**Published:** 2026-02-21

**Authors:** Eenas Ghura, Jan Gaebel, Thomas Neumuth, Andreas Dietz, Gunnar Wichmann, Matthaeus Stoehr

**Affiliations:** 1 Department of Otorhinolaryngology, Head and Neck Surgery, University Hospital Leipzig, 04103 Leipzig, Germany; eenas.ghura@medizin.uni-leipzig.de (E.G.); andreas.dietz@medizin.uni-leipzig.de (A.D.); gunnar.wichmann@medizin.uni-leipzig.de (G.W.); 2 Innovation Center Computer Assisted Surgery (ICCAS), Faculty of Medicine, University Leipzig, 04103 Leipzig, Germany; jan.gaebel@medizin.uni-leipzig.de (J.G.); thomas.neumuth@medizin.uni-leipzig.de (T.N.)

**Keywords:** cutaneous squamous cell carcinoma, cSCC, immunotherapy, molecular tumor board, artificial intelligence, immune checkpoint inhibitors, PD-1 inhibitors, mutational landscape, Bayesian network, clinical decision support system (CDSS)

## Abstract

Background: One of the most prevalent non-melanoma skin cancers (NMSCs) is cutaneous squamous cell carcinoma (cSCC), which is typically treated surgically. For patients with advanced or inoperable disease, systemic therapies—particularly immune checkpoint inhibitors—have become increasingly important. The anti-PD-1 monoclonal antibody Cemiplimab was approved for the treatment of advanced cSCC, providing patients who are unable to receive conventional therapy with additional options. Methods: In this study, we developed a clinical decision support tool based on Bayesian networks (BNs) to help clinicians choose the most suitable treatment strategies for cSCC. The model can manage missing or uncertain data and includes patient-specific clinical, histological, and genetic information, such as tumor type, stage, and PD-L1 expression. Results: Using data from 66 patients with either basal cell carcinoma (BCC) or cSCC, we retrospectively validated the model by comparing the treatment recommendations from the tool with the actual choices made by multidisciplinary tumor boards. The model demonstrated an overall accuracy of 95.5% and statistical significance with a *p*-value of <0.001. Conclusions: Our results suggest that BNs are a valuable tool for representing complex clinical decision-making processes.

## Simple Summary

Treatment decision-making has become increasingly challenging, especially in oncology, due to the growing number of available therapeutic options, particularly in advanced stages of disease. Cutaneous squamous cell carcinoma, one of the most common skin malignancies, is usually treated surgically. However, treatment selection may become more complex in advanced or unresectable cases. In recent years, immune checkpoint inhibition (e.g., Cemiplimab) has extended therapeutic options. In this study, we developed a Bayesian network–based decision support model to assist clinicians in selecting appropriate treatment strategies for patients with cutaneous squamous cell carcinoma.

## 1. Introduction

Cutaneous squamous cell carcinoma (cSCC) is the second most prevalent non-melanoma skin cancer (NMSC), accounting for approximately 20% of cases, whereas basal cell carcinoma (BCC) occurs more frequently (70–80%) [1,2]. In the United States, the annual incidence of cSCC exceeds 1.8 million cases [3]. Most cases are localized, but approximately 40,000 progress to locally advanced or metastatic disease, substantially increasing morbidity [4]. In Germany, NMSC (ICD-10 C44) is not comprehensively recorded due to predominantly outpatient management, limiting epidemiological data [5,6]. Globally, NMSC incidence has risen by 50–200% over the past three decades, with cSCC predominating [1].

Major risk factors include chronic ultraviolet exposure, immunosuppression, and aging. UV radiation induces DNA damage and mutational burden if unrepaired, while immune deficiencies and age-related immune decline further increase cancer risk. However, not all of these factors directly influence treatment selection [5,7,8].

Surgery, if applicable, followed by adjuvant radiotherapy, remains the standard therapy. For inoperable or incompletely resectable tumors, definitive radiotherapy is indicated [9,10,11]. In patients with extensive local or metastatic disease, systemic therapy—primarily immunotherapy—is the main option [12,13,14,15]. Clinical risk factors for recurrence and metastasis include tumor size >2 cm, location on ear, lip, or temple, immunosuppression, perineural invasion, poor differentiation, and fixation to underlying tissue [16,17,18].

Treatment decisions are typically made in multidisciplinary tumor boards, integrating guidelines, clinical trials, real-world evidence, and expert opinion. In selected cases, individualized off-label therapies may be considered [19,20,21]. Immune checkpoint inhibitors targeting PD-1 or PD-L1, such as Cemiplimab and Pembrolizumab, activate T and NK cell-mediated antitumoral responses. CSCC exhibits a high mutational burden, correlating with responsiveness to PD-1 blockade. Cemiplimab is approved for locally advanced or metastatic cSCC when curative local therapy is not feasible [22,23,24,25]. Common adverse events include fatigue, diarrhea, and nausea, while rare but clinically relevant immune-related events, especially endocrine disorders, remain a concern [26,27].

The growing number of parameters critical for therapy—TNM stage, histology, molecular features, and patient-specific factors—creates increasing complexity in decision-making. This makes it challenging to select the optimal treatment, particularly immunotherapy, for individual patients.

Recent studies have demonstrated the potential of hyperspectral imaging combined with machine learning to improve image-based skin cancer classification at the diagnostic level; however, these approaches primarily focus on lesion detection and classification rather than on structured, interpretable support for therapy decision-making in cSCC [28].

Clinical decision support systems (CDSS) can integrate multidimensional data to guide evidence-based, patient-specific treatment [29,30]. Cypko and Stoehr introduced Bayesian network (BN)–based CDSS, initially for laryngeal carcinoma and later extended to other head and neck cancers [31,32].

Here, we describe the development of a BN–based CDSS for cSCC, incorporating patient-specific and tumor-related characteristics to provide transparent, individualized treatment recommendations and support multidisciplinary tumor board decisions.

## 2. Materials and Methods

### 2.1. Literature Review

Initially, a literature review was conducted with the aim of enabling the inclusion of consolidated medical knowledge relating to cSCC’s mutational landscape and implications on the therapeutic strategies involved. Searches in major databases, including MEDLINE via PubMed, Embase, Cochrane Library, and Web of Science, were carried out.

The initial search using the term “cutaneous squamous cell carcinoma survival” yielded a multitude of entries. Further searches were conducted using terms such as “cSCC”, “immunotherapy”, “tumor board”, “molecular tumor board”, “immune checkpoint inhibitors”, “PD-1 inhibitors”, “mutational landscape”, “Bayesian network”, “prognosis”, “AI”, “Cemiplimab”, and “BCC”.

The study considered novel targeted therapies approved for cSCC treatment, such as Cemiplimab, and their impact on treatment decision paradigms, integrating changes in international clinical practice guidelines. Evaluation of indications and usability included examining German medical guidelines for cSCC.

### 2.2. Bayesian Networks

BNs are mathematical frameworks that represent probabilistic relationships among categorical variables in a directed acyclic graph [33]. The variables encompass various states of a real-world concept that reflect potential outcomes. Connections between variables are depicted as edges in the graph, indicating direct dependencies and establishing causal relationships characterized by conditional probability tables (CPT). These tables encode the prior stochastic relationships necessary for estimating the likelihood of one state influencing another. In a medical context, such a model could incorporate clinical observations (such as clinical findings, diagnoses, or histological examination results) from a specific case to create a personalized patient model. Through Bayesian inference algorithms, the model can then assess the probability of unobserved or unobservable variables, such as treatment options for different cancer types. BNs inherit the major advantage of comprehensibility, reproducibility and traceability compared to other machine learning methods. In head and neck malignancies specifically, BNs provide a structured approach to integrating risk factors, clinical findings, and diagnostic results under uncertainty. The joint probability distribution over all variables $X=\{X_{1},\ldots ,\;X_{n}\}$ factorizes into local conditional distributions as $P\left(X_{1},\ldots ,\;X_{n}\right)=\prod_{i}P\left(X_{i}\;\right|\;P_{a}(X_{i}))$ , thus enabling inference. Through Bayesian inference algorithms, the model can then assess the probability of unobserved or unobservable variables, such as treatment options for different cancer types. Inference consists of computing posterior probabilities given observed evidence. For instance, given a tumor variable T and evidence E=e , the posterior probability is obtained via $P(T|E=e)=\frac{P(E=e\;|\;T)\;P(T)}{\sum_{t}P(E=e\;|\;T=t)\;P(T=t)}$ . This formulation updates prior tumor prevalence with patient-specific findings. As another example, consider a BN for suspected laryngeal carcinoma. Let T denote the presence of malignancy, H persistent hoarseness, and I a suspicious lesion on laryngoscopic imaging. Assuming conditional independence of findings given the tumor state, the posterior probability of malignancy is $P\left(T\;\right|\;H=true,\;I=true)=P\left(H\;\right|\;T)\;P\left(I\;\right|\;T)\;P(T)$ , with normalization over T ∈{present, absent} . The resulting posterior can support decisions such as expedited biopsy, imaging escalation, or early oncologic referral.

### 2.3. Model Development

The process for developing a model for skin tumor treatment options (Cemiplimab and/or surgery) is described in this section. We utilized an expert-based modeling approach due to the lack of sufficient patient data to utilize a machine learning approach. Therefore, the graphical model was developed by translating clinical knowledge from existing guidelines, identified studies and current research (see Section 2.1) and significant variables such as tumor type (SCC or BCC), TNM classification, and PD-L1 expression. These variables form the basis of the model.

Modeling was facilitated using the GeNIe software (GeNIe Version 2.2, distributed by Bayesfusion, Druzdzel et al., University of Pittsburgh, PA, USA, https://www.bayesfusion.com/genie/, accessed on 13 March 2018). This software enabled the incorporation of additional properties and references.

Decision nodes were established to represent different treatment options such as Cemiplimab and/or surgery, with respective possible outcomes defined as states to obtain information about underlying dependencies and probabilities.

Clinical information relevant for calculations, such as PD-L1 status and TNM classification and tumor type was modeled as parent nodes influencing decisions. The entire model was thematically organized to ensure clarity and comprehensibility, aimed at supporting the decision-making process for therapy options in cSCC.

### 2.4. Prerequisites for Therapy

This section describes important basic information in order to provide Cemiplimab or surgery to patients who have been diagnosed with cSCC, as determined mainly by the TNM classification, leading to the clinical decision, or in complex cases decision of the tumor board. Without the need for additional selection criteria, these parameters together direct the treatment protocol.

#### 2.4.1. Molecular Tumor Markers

The biomarkers include Tumor Proportion Score (TPS) and Combined Positive Score (CPS) for PD-L1 expression. TPS represents the percentage of PD-L1 positive tumor cells among at least 100 tumor cells (as percentage), while CPS counts for the total number of PD-L1 positive cells among 100 tumor cells. CPS takes into account both the tumor cells and the rest of the tumor microenvironment, whereas TPS only involves the tumor cells. These biomarkers are very useful in tailoring the treatment to suit an individual’s needs and also to assess the effectiveness of the treatment on a patient. To date, the approval of Cemiplimab for the treatment of cSCC and BCC is not contingent upon specific TPS or CPS thresholds, indicating that PD-L1 expression is not a prerequisite for its clinical use.

Still, we included CPS and TPS in our analysis because they may be relevant for demonstrating the ability of BN to handle missing or unknown clinical information when supporting decision-making [34]. Moreover, a meta-analytic study shows that patients with cSCC in the head and neck region and with PD-L1 expression show a superior objective response rate (ORR) when treated with PD-1/PD-L1 inhibitors [35].

#### 2.4.2. Annotation of Probabilities

The process of annotating probabilities within the framework of the CPT necessitates a meticulous representation of contemporary clinical knowledge to facilitate informed clinical judgments. For instance, it is imperative to discern the probability of disease manifestation upon the identification of specific symptomatic presentations, as well as the probability that a particular therapeutic modality is optimally suited for a given patient’s condition.

In populating the CPTs, we relied on established clinical guidelines, FDA approvals, relevant clinical trials, and reputable scientific literature to estimate probabilities for the therapy at different combinations of influencing factors (parental nodes, e.g., disease stages), as exact probabilities for every combination are not explicitly reported in the literature. These initial estimates were further refined through discussions with expert clinicians and tumor board members to ensure that the CPTs reflect both the best available evidence and expert clinical judgment.

Probability assignments were conducted utilizing GeNIe software, which facilitated the portrayal of the likelihood associated with distinct clinical states. These probabilities spanned a spectrum from 1% to 99%, predicated on comprehensive reviews of extant literature. The values serve as an indication of the tendency for which therapy is preferred under standard conditions.

Cemiplimab and surgery are two treatment options that are integrated into our strategy. Based on clinical experience, the model calculates probability values for each target node that range from 1% to 99%. The decision-making process is guided by the model’s prioritization of the option with the highest computed probability, as surgical therapy is typically the primary treatment choice. The therapy with the higher probability is consistently advised, while the treatment with the lower probability is categorized as a subordinate suggestion (i.e., not recommended as the first option). Treatment recommendations are based on the comparison of the probabilities given for the specific treatment option.

### 2.5. Model Verification

A cyclical, expert-informed approach was used to validate the BN model. Certified head and neck oncology specialists, clinical researchers, and computational scientists skilled in probabilistic modeling were all actively involved in the development process. The experts evaluated the model’s structure, including node selection and conditional relationships, as well as the coherence and reliability of assigned probabilities, through a series of structured consultations and iterative feedback sessions. Targeted improvements were made to particular parameter values and interdependencies based on clinical input. Every suggestion was documented and carefully integrated into the model, paying careful attention to both ensuring mathematical soundness and preserving consistency with accepted clinical knowledge.

### 2.6. Model Validation

The model was retrospectively validated to provide a proof-of-concept for the presented BN-based CDSS. We aimed at demonstrating model validation and decision support feasibility, not hypothesis testing of a single parameter.

We used clinical data from 66 cases, some of which were discussed at our multidisciplinary tumor board, while the remaining cases were managed based on clinical decision-making. The cases were selected based on predefined inclusion criteria, specifically patients with cSCC and BCC requiring a treatment decision involving either immunotherapy or surgical intervention.

For each patient, the BN computed posterior probabilities using the Bayesian principle of conditional probabilities with the given patient-specific data and calculating the posterior probabilities using the CPTs. We compared the decisions proposed by the model with those made by the clinical experts or the tumor board. This comparison was conducted by calculating predictive values and ROC analysis.

### 2.7. Patient Cohort

A total of 66 patients were included in the study. They were treated at the University Hospital of Leipzig between 2020 and 2023. The specific data on the retrospectively analyzed dataset including sex, histology (only cSCC and BCC were included), TNM stages, CPS and TPS and treatment are displayed in Table 1.

## 3. Results

### 3.1. The Head and Neck Skin Cancer Model

**Figure 1:**
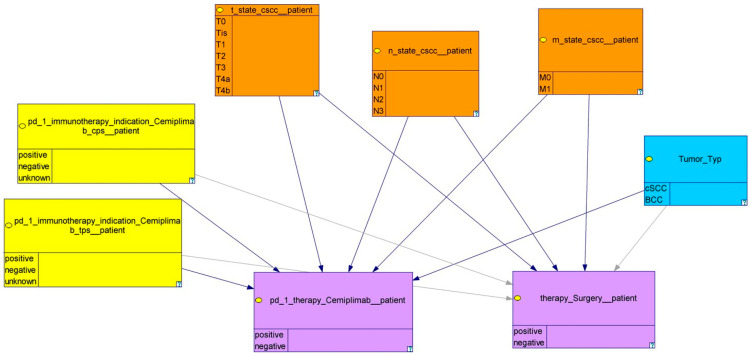
An overview of the model: nodes with the specific information are displayed as colored boxes. Orange: T-, N-, and M-category, yellow: PD-L1 status as CPS and TPS, blue: histologic differentiation, lilac: treatment.

The constructed model comprises eight nodes, systematically organized into subgroups. These subgroups are visually differentiated using color coding and connected by causal dependencies (black arrows), as depicted in Figure 1. To elucidate the functionality of the model, a representative example from one of the subgroups is presented.

### 3.2. Application of the Model

**Figure 2:**
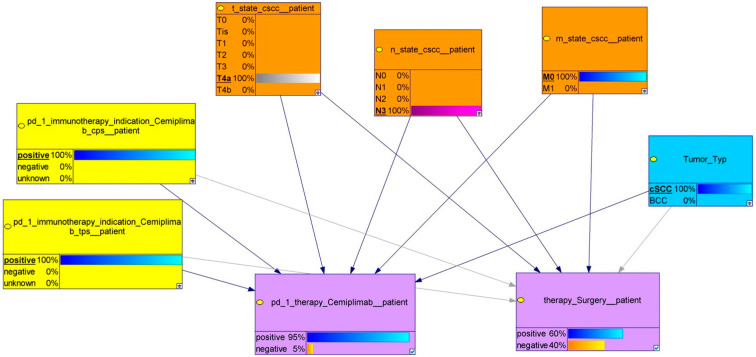
Display of a patient specific model with selected individual information (as marked in the parental knots by underline and bold type, 100%). The combination of the variables leads to a decision whether surgery or Cemiplimab is favorable. In this specific case, treatment with Cemiplimab is estimated to be much more likely (95%) than surgery, which may still be possible (60%).

To demonstrate the practical functioning of the model, we utilized the data of a hypothetical patient with the following characteristics: histology of cSCC: positive, M-stage: M0; T-stage: T4a; N-stage: N3; TPS positive; and CPS: positive. After the inference algorithm estimated the likelihood of the hidden states, our model estimated the chance of surgery at 60% and the effectiveness of Cemiplimab at 95%, as depicted in Figure 2. These predictions are in accordance with current practice guidelines. According to our model and the comparison of probabilities, the first-line treatment recommendation for this hypothetical patient is Cemiplimab.

**Figure 3:**
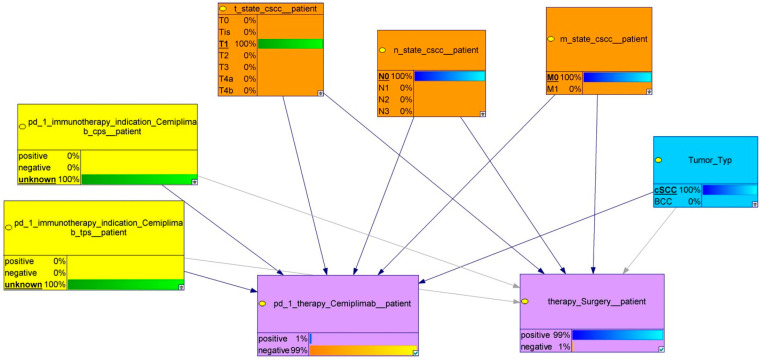
Display of a patient specific model with selected individual information (as marked in the parental knots by underline and bold type, 100%). The combination of the variables leads to a decision whether surgery or Cemiplimab is favorable. In this specific case, treatment with Cemiplimab is practically excluded (1%), while surgery is the treatment of choice (99%).

For patients with cSCC: positive, M-stage: M0; T-stage: T1; N-stage: N0; TPS unknown; and CPS: unknown. The model also predicted a 1% chance of using Cemiplimab for the patient since there are no current recommendations for its use in this particular patient population, as depicted in Figure 3.

### 3.3. Model Performance

The model was further validated through a comparative analysis between its predicted case outcomes and the actual treatments administered. This analysis was based on a dataset of 66 cases that were either discussed in the interdisciplinary tumor board or managed through clinical decision-making at our center.

The sensitivity, specificity, and accuracy for Cemiplimab were 81.8%, 98.2%, and 95.5%, respectively. The sensitivity, specificity, and accuracy for surgery were 100%, 90.1%, and 84.8%, respectively. This results in an overall accuracy of the model considering both nodes together of 95.5%. The trustworthiness of the therapeutic suggestions is supported by the statistical significance of the data (*p* < 0.001).

**Figure 4:**
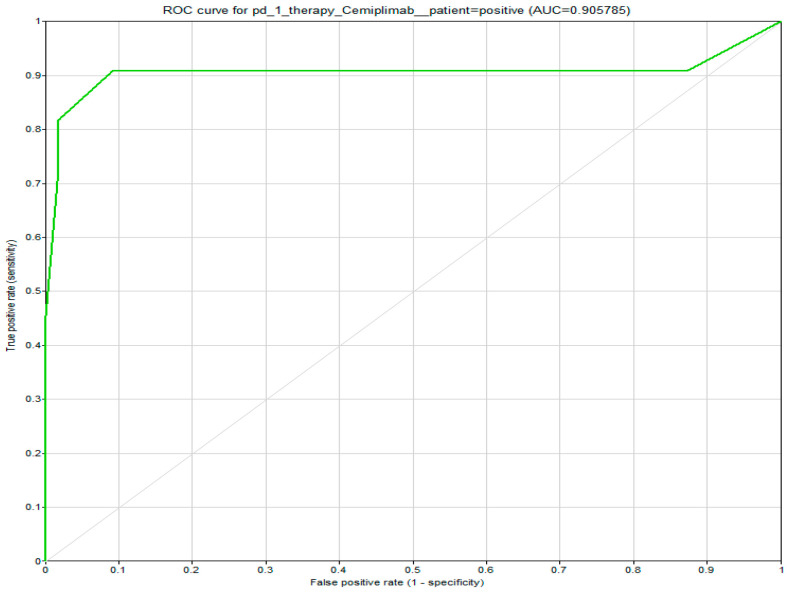
Display of the Receiver Operating Characteristic (ROC) curve illustrating the predictive performance of the model for Cemiplimab = positive with a calculated area under the curve (AUC) of 90.6%.

The model’s performance for Cemiplimab was assessed using Receiver Operating Characteristic (ROC) analysis. The results showed that the Cemiplimab node demonstrated strong predictive accuracy, as depicted in Figure 4.

In 63 cases, the model calculated the identical treatment option based on the given parameters from the dataset compared to the selected treatment according to the patient files; in three cases, however, the recommendations were incongruent (Table 2). These differences were ascribed to particular clinical characteristics that the probabilistic framework of the model could not adequately account for. Deviations from the model’s projected recommendations were primarily caused by the patients’ unique traits, concurrent medical problems, and the location of the tumor.

The first patient (A) with T2 N0 M0, for example, was unable to undergo surgery since the tumor was situated in the ocular angle and nasolacrimal duct, where surgical resection was not functionally feasible. The second patient (B) with T2 N0 M0 could not receive surgical treatment due to severe comorbidities and high risk related to anesthesia and complications, including impaired wound healing.

The third case (C) was more complex and involved a patient with pT3 N2 M0 cSCC and a known history of chronic lymphocytic leukemia (CLL). Application of the decision model showed a higher probability for treatment with Cemiplimab (80%) compared with surgical management (70%), identifying Cemiplimab as the more favorable therapeutic option.

Histopathological examination of the resected lymph nodes following surgery revealed infiltration by both CLL and metastatic SCC. Although adjuvant radiotherapy was recommended by the multidisciplinary tumor board, it could not be administered due to the patient’s poor general condition.

Approximately three months after surgery, the patient developed a locoregional nodal recurrence, at which point systemic immunotherapy was initiated, and treatment with Cemiplimab was commenced.

These circumstances only became apparent during the in-depth follow-up investigation and were not available at the time of initial clinical assessment. Despite the absence of this information in the primary dataset, the model was still able to suggest the appropriate treatment.

## 4. Discussion

In this study, we introduce a modeling approach that demonstrated satisfactory performance for calculating treatment decisions to distinguish between surgical treatment and immunotherapy with Cemiplimab for cases with NMSC. This may potentially support physicians in their decision-making process after prospective testing and clinical introduction. We used clinical data and received medical recommendations from tumor specialists to quantify the likelihood of different treatment choices using a BN. Our model’s assessment both identifies areas that need improvement and demonstrates its potential for clinical decision support.

Compared with many currently available models—whose major limitation in clinical practice is the algorithmic “black-box syndrome,” characterized by the inability of clinicians to understand how outputs are generated or to trace the basis of even accurate predictions—BNs offer inherent reproducibility and traceability [36].

Machine learning approaches often require large amounts of high-quality, labeled data, which can be difficult to obtain in clinical settings. Rule-based systems are easy to understand but can become complex, require constant updates, and depend on specific input formats [37].

A scoping review on the use of BNs for disease prognosis and prediction reported that BN models achieve predictive performance at least comparable to commonly used machine learning and statistical methods, such as logistic regression (LR), support vector machines (SVM), neural networks (NN), and decision trees (DT), while offering further benefits in terms of interpretability and handling of missing data [34].

The model has a high overall accuracy of 95.5%, identifying the correct treatment option in 63 of 66 cases. This outcome shows the potential usefulness of probabilistic frameworks for assisting clinicians in making judgments, particularly in complicated cancer cases. The model bases its recommendations on evaluating the probabilities of each treatment option individually, with the therapy having the highest probability designated as the primary choice. Although surgical intervention is considered the gold standard and is frequently preferred, the model assigned clear priority to Cemiplimab in cases where its calculated probability of treatment recommendation exceeded that of surgery. This method shows how the model may incorporate several clinical aspects and offer case-specific, individualized treatment recommendations.

Sensitivity and specificity analyses showed that the two treatment nodes performed differently. Sensitivity, specificity, and accuracy were 100%, 90.1%, and 84.8% for surgery, and 81.8%, 98.2%, and 95.5% for Cemiplimab, respectively. The imbalance in the dataset is reflected in the lower specificity for surgery, which highlights the significance of assessing both treatment nodes together. Decision-making in clinical practice should be based on the treatment with the higher calculated probability, with the model serving as a support tool rather than the sole determinant.

Deviations from the model’s predictions occurred in three cases. These discrepancies were primarily attributable to individual patient characteristics that the probabilistic framework could not fully capture, including atypical tumor localization, comorbidities, and other clinical nuances. For example, certain patients were not suitable for surgical intervention due to comorbid conditions, while others presented with tumor locations favoring alternative therapeutic approaches. These cases highlight that, despite high predictive accuracy, clinical judgment remains essential, particularly for patients with complex or atypical presentations, in particular comorbidities and high risk for complications.

Although the model reasonably captures the clinical decision-making process, further improvements are possible, particularly by incorporating additional clinical parameters. However, adding numerous dependencies to the model leads to an exponential increase in the number of conditional probability tables, which complicates model development and may limit computational feasibility. Furthermore, the model’s prediction ability is limited by the absence of trustworthy and complete clinical data, and it is extremely difficult to incorporate unclear or insufficient data. That is why we refrained from updating the model’s structure to include additional facets, e.g., comorbidities or other clinical nuances. However, the current framework highlights the promising role of probabilistic models in assisting clinical decision-making and shows strong predictive ability.

Due to the specific patient cohort at University Hospital of Leipzig, only a limited number of patients was available for model validation, particularly for less commonly used treatments such as Cemiplimab. Expanding the patient sample may therefore enhance predictive accuracy and improve generalizability to real-world clinical settings. 66 cases are still sufficient to show stable inference behavior and non-random predictive performance, not definitive clinical superiority. Bayesian models also explicitly incorporate expert knowledge or literature, which stabilizes learning in small datasets. This prior information constrains the hypothesis space and mitigates overfitting, enabling meaningful inference even in relatively small cohorts.

Previous studies have reported comparable findings. Based on a cohort of 25 patients, Huehn et al. developed a BN-based model to assist with immunotherapy decision-making for recurrent and metastatic head and neck squamous cell carcinoma (R/M HNSCC), achieving an accuracy of 84% [38]. Similarly, Cypko et al. developed a model with 100% predictive accuracy focusing on TNM classification for laryngeal cancer [31].

The long-term goal is to develop a large model that can be integrated into clinical software systems to automatically generate evidence-based treatment recommendations directly from electronic patient records, without the need for manual data entry. At this stage, patient preferences are not incorporated, as the focus is on delivering scientifically sound, guideline-concordant recommendations. Incorporating such models into a comprehensive BN framework could be highly beneficial for optimizing individualized treatment choices, especially given the increasing number of therapeutic options and the rising incidence of head and neck cancers. The presented cSCC model demonstrated comparable performance in suggesting favorable treatment options relative to previous BN models. In light of these findings, we recommend that additional models be developed and validated to support clinical decision-making in the future. Ultimately, integrating different models into a general patient decision-support system may improve individualized treatment planning and, through evidence-based decision-making, enhance clinical outcomes.

## 5. Conclusions

Digital decision-support systems may offer a feasible option to standardize treatment decisions and traceability for personalized therapy strategies. We present a BN model that supports the decision-making process based on tumor boards and clinical decision-making when determining therapeutic options for patients with cSCC, using clinical and molecular data to select optimal treatments in an individualized manner. Our results indicate that BNs are a promising tool for mapping complex decision-making processes in a comprehensible and evidence-based way. The model provides a structured representation of clinical reasoning, enhancing the transparency and consistency of therapeutic decisions. As demonstrated, the model is able to accurately aid in decision-making and provides a foundation for more personalized therapies. As adding new nodes and edges for newly introduced diagnostics and inclusion/exclusion criteria for emerging therapies addressing specific molecular targets is easy, using such an expanded variant of our BN model would enable it to keep pace with requirements in future decision-making processes.

## Figures and Tables

**Table 1:** Patient Cohort Characteristics. cSCC: cutaneous squamous cell carcinoma; BCC: basal cell carcinoma; T category (Tumor stage, AJCC/UICC 8th edition, 2017): Tx = primary tumor cannot be assessed, T0 = no evidence of primary tumor, Tis = carcinoma in situ, T1–T4 = increasing tumor size or local invasion; N category (Regional lymph nodes): Nx = regional lymph nodes cannot be assessed, N0 = no regional lymph node metastasis, N1–N3 = increasing nodal involvement; M category (Distant metastasis): Mx = distant metastasis cannot be assessed, M0 = no distant metastasis, M1 = distant metastasis present; PD-L1 CPS: Combined Positive Score; PD-L1 TPS: Tumor Proportion Score.

Variable	Category	N	%
Sex	Male	45	68.2%
	Female	21	31.8%
Histology	cSCC	22	33.3%
	BCC	44	66.7%
Tumor stage (T category)	Tx	1	1.5%
	T0	3	4.6%
	Tis	4	6.1%
	T1	46	69.7%
	T2	5	7.6%
	T3	4	6.1%
	T4a	2	3.0%
	T4b	1	1.5%
Nodal status (N category)	Nx	48	72.7%
	N0	11	16.7%
	N2	1	1.5%
	N3	6	9.1%
Distant metastasis	Mx	45	68.2%
(M category)	M0	20	30.3%
	M1	1	1.5%
PD-L1 (CPS)	Positive	7	10.6%
	Negative	0	0.0%
	Unknown	59	89.4%
PD-L1 (TPS)	Positive	5	7.6%
	Negative	2	3.0%
	Unknown	59	89.4%
Therapy	Cemiplimab	11	16.7%
	Surgery	55	83.3%

**Table 2:** Patients with incorrect treatment recommendations.

Parameter	Patient A	Patient B	Patient C
Tumor Type	cSCC	cSCC	cSCC
T	2	2	3
N	0	0	2
M	0	0	0
Surgery	No	No	Yes
CPS	Unknown	Positive	Unknown
TPS	Unknown	Negative	Unknown
Cemiplimab	Yes	Yes	No
Model for Surgery (%)	90%	90%	70%
Matches Model—Result? (Surgery)	No	No	No
Model for Cemiplimab (%)	5%	5%	80%
Matches Model—Result? (Cemiplimab)	No	No	No
Comparison: Model vs. Actual Outcome	No	No	No

## Data Availability

Data is contained within the manuscript. Further information about the data may be provided on request.

## List of Abbreviations

AI	Artificial Intelligence
AUC	Area under the Curve
BCC	Basal Cell Carcinoma
BN	Bayesian Network
CDSS	Clinical Decision Support System
CLL	Chronic Lymphocytic Leukemia
CPS	Combined Positive Score
cSCC	Cutaneous Squamous Cell Carcinoma
CPT	Conditional Probability Tables
DT	Decision Trees
FDA	Food and Drug Administration
ICI	Immune Checkpoint Inhibitor
LR	Logistic Regression
NMSC	Non-Melanoma Skin Cancer
NN	Neural Networks
PD-1	Programmed Cell Death Protein 1
PD-L1	Programmed Cell Death Ligand 1
PNI	Perineural Invasion
ROC	Receiver Operating Characteristic
R/M HNSCC	Recurrent and Metastatic Head and Neck Squamous Cell Carcinoma
SVM	Support Vector Machines
TPS	Tumor Proportion Score
TNM	Tumor, Node, Metastasis
UV	Ultraviolet

## References

[1] Zhang Y., Guo Z., Wang H., Li B. (2023). Global Status of Research on Cutaneous Squamous Cell Carcinoma and Its Programmed Cell Death: Bibliometric and Visual Analysis from 2012 to Middle 2022. Front. Oncol..

[2] Leiter U., Eigentler T., Garbe C. (2014). Epidemiology of Skin Cancer. Sunlight, Vitamin D and Skin Cancer.

[3] Burns C., Kubicki S., Nguyen Q.-B., Aboul-Fettouh N., Wilmas K.M., Chen O.M., Doan H.Q., Silapunt S., Migden M.R. (2022). Advances in Cutaneous Squamous Cell Carcinoma Management. Cancers.

[4] Grammatica A., Tomasoni M., Fior M., Ulaj E., Gualtieri T., Bossi P., Battocchio S., Lombardi D., Deganello A., Mattavelli D. (2022). Regional Disease in Head and Neck Cutaneous Squamous Cell Carcinoma: The Role of Primary Tumor Characteristics and Number of Nodal Metastases. Eur. Arch. Otorhinolaryngol..

[5] Leiter U., Heppt M.V., Steeb T., Alter M., Amaral T., Bauer A., Bechara F.G., Becker J.C., Breitbart E.W., Breuninger H. (2023). S3-Leitlinie “Aktinische Keratose Und Plattenepithelkarzinom Der Haut”—Update 2023, Teil 2: Epidemiologie Und Ätiologie, Diagnostik, Therapie Des Invasiven Plattenepithelkarzinoms Der Haut, Nachsorge Und Prävention: S3 Guideline „actinic Keratosis and Cutaneous Squamous Cell Carcinoma”—Update 2023, Part 2: Epidemiology and Etiology, Diagnostics, Surgical and Systemic Treatment of Cutaneous Squamous Cell Carcinoma (cSCC), Surveillance and Prevention. J. Dtsch. Dermatol. Ges..

[6] Leiter U., Garbe C., Reichrath J. (2008). Epidemiology of Melanoma and Nonmelanoma Skin Cancer—The Role of Sunlight. Sunlight, Vitamin D and Skin Cancer.

[7] Jiang R., Fritz M., Que S.K.T. (2024). Cutaneous Squamous Cell Carcinoma: An Updated Review. Cancers.

[8] Kaufhold M., Asadi S., Ghoreishi Y., Brekner A., Grabbe S., Stege H., Nassabi H. (2025). Cutaneous Squamous Cell Carcinoma Risk Factors: Are Current Criteria Still Valid? A Retrospective, Monocenter Analysis. Life.

[9] Mendenhall W.M., Amdur R.J., Hinerman R.W., Cognetta A.B., Mendenhall N.P. (2009). Radiotherapy for Cutaneous Squamous and Basal Cell Carcinomas of the Head and Neck. Laryngoscope.

[10] Maubec E. (2020). Update of the Management of Cutaneous Squamous-Cell Carcinoma. Acta Derm. Venereol..

[11] Likhacheva A., Awan M., Barker C.A., Bhatnagar A., Bradfield L., Brady M.S., Buzurovic I., Geiger J.L., Parvathaneni U., Zaky S. (2020). Definitive and Postoperative Radiation Therapy for Basal and Squamous Cell Cancers of the Skin: Executive Summary of an American Society for Radiation Oncology Clinical Practice Guideline. Pract. Radiat. Oncol..

[12] Mager L., Gardeen S., Carr D.R., Shahwan K.T. (2023). Cemiplimab for the Treatment of Advanced Cutaneous Squamous Cell Carcinoma: Appropriate Patient Selection and Perspectives. Clin. Cosmet. Investig. Dermatol.

[13] Hillen U., Leiter U., Haase S., Kaufmann R., Becker J., Gutzmer R., Terheyden P., Krause-Bergmann A., Schulze H.-J., Hassel J. (2018). Advanced Cutaneous Squamous Cell Carcinoma: A Retrospective Analysis of Patient Profiles and Treatment Patterns—Results of a Non-Interventional Study of the DeCOG. Eur. J. Cancer.

[14] Jarkowski A., Hare R., Loud P., Skitzki J.J., Kane J.M., May K.S., Zeitouni N.C., Nestico J., Vona K.L., Groman A. (2016). Systemic Therapy in Advanced Cutaneous Squamous Cell Carcinoma (CSCC): The Roswell Park Experience and a Review of the Literature. Am. J. Clin. Oncol..

[15] Cranmer L.D., Engelhardt C., Morgan S.S. (2010). Treatment of Unresectable and Metastatic Cutaneous Squamous Cell Carcinoma. Oncologist.

[16] Thompson A.K., Kelley B.F., Prokop L.J., Murad M.H., Baum C.L. (2016). Risk Factors for Cutaneous Squamous Cell Carcinoma Recurrence, Metastasis, and Disease-Specific Death: A Systematic Review and Meta-Analysis. JAMA Dermatol..

[17] Crüts E.C., Moermans M.M.G., Abdul Hamid M., Nelemans P.J., Mosterd K. (2024). Perineural Invasion for Risk Stratification in Cutaneous Squamous Cell Carcinoma: A Scoping Review. Dermatology.

[18] Stratigos A.J., Garbe C., Dessinioti C., Lebbe C., Van Akkooi A., Bataille V., Bastholt L., Dreno B., Dummer R., Fargnoli M.C. (2023). European Consensus-Based Interdisciplinary Guideline for Invasive Cutaneous Squamous Cell Carcinoma. Part 1: Diagnostics and Prevention–Update 2023. Eur. J. Cancer.

[19] Specchia M.L., Frisicale E.M., Carini E., Di Pilla A., Cappa D., Barbara A., Ricciardi W., Damiani G. (2020). The Impact of Tumor Board on Cancer Care: Evidence from an Umbrella Review. BMC Health Serv. Res..

[20] Hoefflin R., Geißler A.-L., Fritsch R., Claus R., Wehrle J., Metzger P., Reiser M., Mehmed L., Fauth L., Heiland D.H. (2018). Personalized Clinical Decision Making Through Implementation of a Molecular Tumor Board: A German Single-Center Experience. JCO Precis. Oncol..

[21] Jerusalem G., Coucke P. (2011). The role of multidisciplinary tumor board discussions in treatment decisions. Rev. Med. Liege.

[22] Fritz J.M., Lenardo M.J. (2019). Development of Immune Checkpoint Therapy for Cancer. J. Exp. Med..

[23] Migden M.R., Rischin D., Schmults C.D., Guminski A., Hauschild A., Lewis K.D., Chung C.H., Hernandez-Aya L., Lim A.M., Chang A.L.S. (2018). PD-1 Blockade with Cemiplimab in Advanced Cutaneous Squamous-Cell Carcinoma. N. Engl. J.Med..

[24] Gross N.D., Miller D.M., Khushalani N.I., Divi V., Ruiz E.S., Lipson E.J., Meier F., Su Y.B., Swiecicki P.L., Atlas J. (2022). Neoadjuvant Cemiplimab for Stage II to IV Cutaneous Squamous-Cell Carcinoma. N. Engl. J. Med..

[25] Migden M.R., Khushalani N.I., Chang A.L.S., Lewis K.D., Schmults C.D., Hernandez-Aya L., Meier F., Schadendorf D., Guminski A., Hauschild A. (2020). Cemiplimab in Locally Advanced Cutaneous Squamous Cell Carcinoma: Results from an Open-Label, Phase 2, Single-Arm Trial. Lancet Oncol..

[26] Rischin D., Migden M.R., Lim A.M., Schmults C.D., Khushalani N.I., Hughes B.G.M., Schadendorf D., Dunn L.A., Hernandez-Aya L., Chang A.L.S. (2020). Phase 2 Study of Cemiplimab in Patients with Metastatic Cutaneous Squamous Cell Carcinoma: Primary Analysis of Fixed-Dosing, Long-Term Outcome of Weight-Based Dosing. J. Immunother. Cancer.

[27] Zhan L., Feng H., Liu H., Guo L., Chen C., Yao X., Sun S. (2021). Immune Checkpoint Inhibitors-Related Thyroid Dysfunction: Epidemiology, Clinical Presentation, Possible Pathogenesis, and Management. Front. Endocrinol..

[28] Lin T.-L., Mukundan A., Karmakar R., Avala P., Chang W.Y., Wang H.C. (2025). Hyperspectral imaging for enhanced skin cancer classification using machine learning. Bioengineering.

[29] Pawloski P.A., Brooks G.A., Nielsen M.E., Olson-Bullis B.A. (2019). A Systematic Review of Clinical Decision Support Systems for Clinical Oncology Practice. J. Natl. Compr. Cancer Netw..

[30] Beauchemin M., Murray M.T., Sung L., Hershman D.L., Weng C., Schnall R. (2019). Clinical Decision Support for Therapeutic Decision-Making in Cancer: A Systematic Review. Int. J. Med. Inform..

[31] Cypko M.A., Stoehr M. (2019). Digital Patient Models Based on Bayesian Networks for Clinical Treatment Decision Support. Minim. Invasive Ther. Allied Technol..

[32] Hikal A., Gaebel J., Neumuth T., Dietz A., Stoehr M. (2023). A Treatment Decision Support Model for Laryngeal Cancer Based on Bayesian Networks. Biomedicines.

[33] Pearl J. (1988). Probabilistic Reasoning in Intelligent Systems: Networks of Plausible Inference.

[34] Polotskaya K., Muñoz-Valencia C.S., Rabasa A., Quesada-Rico J.A., Orozco-Beltrán D., Barber X. (2024). Bayesian Networks for the Diagnosis and Prognosis of Diseases: A Scoping Review. Mach. Learn. Knowl. Extr..

[35] Paderno A., Petrelli F., Lorini L., Capriotti V., Gurizzan C., Bossi P. (2024). The Predictive Role of PD-L1 in Head and Neck Cancer: A Systematic Review and Meta-Analysis. Oral Oncol..

[36] Agard G., Roman C., Guervilly C., Ouladsine M., Boyer L., Hraiech S. (2025). Improving Sepsis Prediction in the ICU with Explainable Artificial Intelligence: The Promise of Bayesian Networks. J. Clin. Med..

[37] Tschochohei M., Adams L.C., Bressem K.K., Lammert J. (2025). KI-gestützte klinische Entscheidungsunterstützungssysteme: Herausforderungen und Potenziale [AI-enabled clinical decision support systems: Challenges and opportunities]. Bundesgesundheitsblatt Gesundheitsforsch. Gesundheitsschutz.

[38] Huehn M., Gaebel J., Oeser A., Dietz A., Neumuth T., Wichmann G., Stoehr M. (2021). Bayesian Networks to Support Decision-Making for Immune-Checkpoint Blockade in Recurrent/Metastatic (R/M) Head and Neck Squamous Cell Carcinoma (HNSCC). Cancers.

